# Plexin A2 Knockdown Enhances Apoptosis in Chemotherapy Treated Melanoma Cells

**DOI:** 10.32604/or.2026.069234

**Published:** 2026-04-22

**Authors:** Nadezhda Palkina, Aleksandra Esimbekova, Ekaterina Lapkina, Victoriia Kutsenko, Ivan Zinchenko, Egor Dereviankin, Elena Anisimova, Andrei Savchenko, Tatiana Ruksha

**Affiliations:** 1 Department of Pathophysiology, Krasnoyarsk State Medical University, Krasnoyarsk, Russia; 2 Laboratory of Cell Molecular Physiology and Pathology, Federal Research Center, Krasnoyarsk Science Center of the Siberian Branch of the Russian Academy of Sciences, Krasnoyarsk, Russia

**Keywords:** Apoptosis, chemoresistance, melanoma, plexin A2 (PLXNA2), quiescence

## Abstract

**Background:**

Cancer cells are characterized by the ability to exit reversibly from the cell cycle to resist an unfavorable environment. This study elucidates alterations in adhesion molecule expression in melanoma cells acquiring resistance to dacarbazine (DTIC) and entering the G0 state. Plexin A2 (PLXNA2) was identified as a focal adhesion-related molecule implicated in carcinogenesis.

**Methods:**

Applying siRNA-mediated knockdown, the effects of altered *PLXNA2* expression in melanoma cells were evaluated. *PLXNA2* expression was determined by real-time quantitative reverse transcription PCR, immunoblotting, and immunocytochemistry. Cell cycle phase distribution among dacarbazine-treated cells and their apoptosis levels were quantified by flow cytometry, while adhesion to fibronectin was evaluated spectrophotometrically.

**Results:**

Our findings indicated that DTIC treatment modulates melanoma cell interactions with the extracellular matrix, facilitating adhesion to collagen IV, fibronectin, and laminin. Concurrently, integrin expression diminishes upon DTIC exposure. Delete Crucially, focal adhesion signaling molecules, including PLXNA2, Phosphoinositide-3-Kinase Regulatory Subunit 1, and Fibroblast Growth Factor Receptor 2, exhibit increased expression. *PLXNA2* knockdown in DTIC-treated melanoma cells did not affect the percentage of cells residing in the G0 phase of the cell cycle. However, it induced apoptosis in DTIC-treated SK-MEL-2 and A375 melanoma cells and G1 cell cycle arrest in A375 melanoma cells.

**Conclusions:**

These findings suggest that PLXNA2 down-regulation in DTIC-treated cancer cells promotes their apoptosis. Therefore, targeting focal adhesion molecules during chemotherapy can increase the sensitivity of tumor cells to anticancer treatment.

## 1 Introduction

Tumor heterogeneity is intimately related to cancer cell plasticity and drug resistance [1–3]. Diverse cancer cell subpopulations with varying cell-cycle dynamics demonstrate the ability of cancer cells to exit from the cell cycle to a quiescent, non-proliferative state. It has been shown that disseminated cancer cells as well as circulating cancer cells, are predominantly quiescent. Additionally, quiescence is a process that promotes tumor dormancy [4,5].

Tumor dormancy represents a unique biological phenomenon when tumor cells temporarily withdraw from the cell cycle, entering a resting phase known as G0 [6]. During dormancy, cancer cells can exhibit resistance to certain forms of antitumor therapy over extended periods, and dormant cells restarting their activity leads to the recurrence of the disease in its later stages [7].

On the other hand, cell cycle dynamics is tightly related to cell adhesion. Indeed, cell division is dependent on cell contacts with the extracellular matrix. Thus, to enter into the G1 phase of the cell cycle, a cell activates adhesion signaling to start DNA replication [8,9]. Conversely, focal adhesion proteins are involved in apoptosis-inducing events [10,11]. However, quiescent cancer cells are characterized by an altered focal adhesion pattern [12]. Moreover, disseminated cancer cells that are detached from primary tumors and reside in distant organs in a quiescent state until reactivated to start metastasis formation are characterized by dynamical sensing of the extracellular matrix to get external stimuli for survival. It has been reported that quiescent disseminated cancer cells obtain signals for reactivation via contact with the extracellular matrix [13].

In the context of carcinogenesis, cell quiescence is implicated in two critical events: metastasis and drug resistance. These two processes are intricately linked, as disseminated cancer cells frequently exhibit resistance to conventional anticancer therapies [7]. Indeed, the ability of cancer cells to enter into a quiescent state following dissemination may contribute to their survival and persistence in distant sites, ultimately leading to the development of drug-resistant metastatic lesions [14]. Understanding the mechanisms that govern cancer cell quiescence and its impact on drug resistance is crucial for developing more effective therapeutic strategies to prevent and treat metastatic disease.

Non-proliferating cancer cells are characterized by drug resistance due to their reduced antigen pattern and non-proliferative state [15,16]. The mechanisms underlying the drug resistance of quiescent cells are still not clear. As quiescence can be triggered by hypoxia and nutrient insufficiency, the genes associated with these processes were shown to be upregulated both in quiescent and therapy-resistant cancer cells. Moreover, the microenvironment of quiescent cancer cells is reach of M2-type tumor-associated macrophages that leads to T-cell-mediated immunity down-regulation [17].

Quiescent cancer cells residing in distant organs can be reactivated to re-enter the cell cycle and start proliferation, forming clinically visible metastasis [18]. Emerging evidence suggests that the transition to and maintenance of the dormant state are also influenced by a complex interplay of cytokine signaling. Notably, TGF-β2 has been implicated in the regulation of cellular quiescence and dormancy. Specifically, TGF-β2 signaling has been shown to induce cell cycle arrest and promote a dormant phenotype in various cancer types [19]. Although factors triggering the shift to a proliferative state remain unclear, it is evident that cancer cell interactions with the extracellular matrix are crucial for maintaining the quiescent or proliferative state of a cell [20]. Our previous studies showed that the transition of melanoma cells to the G0 phase of the cell cycle under DTIC treatment was associated with the enhancement of their adhesive capacity [12]. Therefore, modulating contacts between cancer cells of various phenotypes and the extracellular matrix can be a novel therapeutic solution for controlling dormant tumors.

Plexins, a family of transmembrane surface proteins that interact with semaphorins, mediate intercellular and cell-matrix communications. These proteins are characterized as regulators of neuronal development and plasticity [21]. When semaphorins bind to plexins, the intracellular domain of a plexin is stimulated which, in turn, triggers GTPase-dependent activation leading to the cytoskeleton rearrangement and conformational activation of integrins—proteins that directly interact with the extracellular matrix [22]. Indeed, plexins were found to be implicated in cancer cell migration [23], immune cell activation [24], and the prometastatic reprogramming of colorectal cancer cells [25].

DTIC belongs to monofunctional alkylating agents which have been applied for cancer treatment for several decades [26]. DTIC is characterized by a low response rate, currently applied as a baseline to evaluate the efficacy of novel anticancer agents. It is important to note that our study does not aim to enhance the activity of DTIC nor to propose its reintroduction into clinical practice. Given its limited efficacy and the increased usage of more effective therapies such as immunotherapies and BRAF inhibitors, DTIC is currently used only in specific circumstances [27,28]. Instead, we leveraged DTIC’s limited effectiveness to generate a population of treatment-resistant, surviving melanoma cells enriched in the G0 phase of the cell cycle. This G0-positive population, induced by DTIC exposure, served as our primary object of investigation, enabling us to explore the mechanisms underlying drug resistance and dormancy in melanoma. Therefore, the present study describes phenotypic features of DTIC-treated melanoma cells under *PLXNA2* knockdown to explore how plexins are involved in cancer cell plasticity, drug resistance, and tumor dissemination. Our focus is on understanding the biology of these surviving cells, rather than on improving DTIC’s direct cytotoxic effects. Consistent with our approach, other studies have also used chemotherapeutic agents such as 5-fluorouracil and temozolomide to induce dormancy and resistance in cancer cells, subsequently using these survivor populations as models to investigate the underlying mechanisms of these phenomena. For example, studies of 5-fluorouracil-induced dormant colon cancer cells revealed the intracellular signaling mechanisms associated with survival [29]. Similarly, temozolomide-resistant glioma cells have been generated and characterized to identify novel therapeutic targets for overcoming drug resistance [30]. These reports, like ours, recognize the value of using chemotherapeutics as experimental models to create cellular models that recapitulate key aspects of drug resistance and dormancy, allowing for a deeper understanding of these complex processes.

## 2 Materials and Methods

For the full name, manufacturer, catalog number, and other information on equipment and reagents, see the Appendix A section.

### 2.1 Cell Culture and Exposure to DTIC

The study used two melanoma cell lines: SK-MEL-2, purchased from BioLot (Moscow, Russia), and A375, provided by the Research Institute of Experimental Diagnostics and Therapy of Tumors (Moscow, Russia).

The selection of the aforementioned cell lines was determined by a number of factors: ATCC® certification, adhesive properties, and differences in their constitutional characteristics, such as proliferation dynamics, migration levels, and invasion, which together mimic the actual heterogeneity of tumors.

Melanoma cells were cultured in DMEM culture medium with glutamine 0.862 g/L and glucose 4.5 g/L (PanEco, Moscow, Russia), containing 10% FBS (Cytiva, Marlborough, MA, USA) and 1% antibiotic-antimycotic (PanEco, Moscow, Russia) in a CO_2_ incubator MSO-5AC CO_2_ at a temperature of 37°C and supply of humid air containing CO_2_ in concentration 5%. All cell lines have been authenticated by STR profiling and the absence of mycoplasma contamination in all cell lines was confirmed using the BioMaster Myco-visor kit PCR detection kit. Cells were subjected to DTIC at a final concentration of 1.2 mmol/L [31]. For this purpose, cells that had undergone at least three passages were grown until the coverage density of the culture container was 80%–90%, then a solution of DTIC in DMSO was added at a concentration of half-maximal inhibition (IC_50_). In the Control, instead of the DTIC solution, DMSO was added at a final concentration of 1%. The cells were incubated for 72 h at 37°C in a CO_2_ incubator, stirring gently on a shaker daily, then the culture medium was changed to fresh and incubated for another 48 h.

### 2.2 Melanoma Cell Adhesion Assay

Melanoma cell adhesion to collagen types I, II, IV, fibronectin, tenascin and vitronectin under DTIC treatment was performed using the ECM Cell Adhesion Array Kit. To study adhesion, suspensions of SK-MEL-2 melanoma cells were prepared at a concentration of 1 × 10^6^ in the kit’s buffer solution and were added to the plate wells pre-rehydrated with phosphate buffer in a volume of 200 μL. The plate was incubated with melanoma cells for 2.5 h. During this time, cells expressing adhesion molecules attached to the bottom of the corresponding wells, while those not expressing them remained unattached. After incubation, the contents of the wells along with the unattached cells were removed and the wells were thoroughly washed, after which the remaining attached cells were fixed and air-dried at room temperature, after which they were lysed and the optical density of each lysate was determined on an EFOS-9305 spectrophotometer at a wavelength of 570 nm. The level of the obtained optical density was taken to correspond to the level of adhesion to one or another component of the extracellular matrix.

To estimate the adhesion to fibronectin in melanoma cells treated with DTIC and knockdown for PLXNA2, we assessed their ability to bind specifically to this component of the extracellular matrix. We created a fibronectin scaffold in the wells of a culture plate. For this purpose, fibronectin lyophilizate was used and a solution was prepared at a concentration of 100 μg/mL, then 30 μL of the resulting solution was added to each well. The next day, cell suspensions were incubated for 2.5 h in a CO_2_ incubator. After incubation, the medium was removed from the wells along with unattached cells that did not adhere to fibronectin, and the monolayer was stained with 150 μL of methylene blue solution, then lysed in 150 μL of DMSO. The intensity of the color of the lysates was assessed using a spectrophotometer at a wavelength of 620–660 nm. The level of obtained optical density was considered to correspond to the level of adhesion to fibronectin.

### 2.3 Knockdown of PLXNA2 Using siRNA

PLXNA2 knockdown was performed by using siRNA. Designed sequences of siRNA for *PLXNA2* were characterized by a binding site corresponding to the sense strand of the siRNA. In addition, we took into account such factors as the length of the sequence (no more than 25 nucleotides), that the content of G/C nucleotides should be in the range of 35%–55%, that the 5^′^ end of the siRNA antisense strand should contain more than A/U-nucleotides, that the complementarity of the siRNA with other transcripts should not exceed 16 nucleotides in length, that the siRNA should not contain repeats of nucleotide motifs and a large number of identical nucleotides in a row (more than 3), and finally, that since it is known that the siRNA strand that has a less thermodynamically stable 5^′^ end binds more efficiently to the RISC complex, an unpaired deoxydinucleotide should be added to the 3^′^ end of each siRNA strand to increase the stability of the duplex and the efficiency of siRNA loading into the RISC complex.

PLXNA2-specific siRNA and its corresponding negative Control (NC) were designed and synthesized by DNA synthesis (Moscow, Russia). To select a target siRNA sequence for the experiment, we used the computational tool BLAST (Basic Local Alignment Search Tool, BLAST, version 2.16.0), which screened the initially generated putative siRNA sequences for potential binding sites with other genes. We ultimately selected the final sequence based on the lowest probability of off-target effects among the remaining sequence variants.

The sequences for *PLXNA2* were GGU AGU GAG UGA UGC UCC UGA UCU A-3^′^ 3^′^-CCA UCA CUC ACU ACG AGG ACU AGA U, as well as the sequence of scrambled siRNA, which is a set of nucleotides in a random sequence, which is a Negative Control (NC): CGA AGU UGG AGU CUG CUG UUA GUC A-3^′^ 3^′^-GCU UCA ACC UCA GAC GAC AAUCAG U. Transfection of siRNA to PLXNA2 and Negative Control into melanoma cells of the SK-MEL-2 and A375 lines was carried out after exposure to the cytostatic drug DTIC according to a previously developed protocol. For 1 mL of cell suspension at a concentration of 4 × 10^4^, 40 pmol of a solution of siRNA to PLXNA2 and Negative Control and 1 μL of a transfection reagent based on lipofectamine GenJect Transfection Reagent were used. Transfection was carried out for 48 h. The effectiveness of suppression of *PLXNA2* expression in DTIC-treated melanoma cells was assessed at various stages of gene expression—at the mRNA level using real-time quantitative Reverse Transcription-PCR (qRT-PCR), and at the protein level using Western blotting and immunohistochemical studies.

### 2.4 Immunoblotting

DTIC-treated SK-MEL-2 and A375 melanoma cells with *PLXNA2* knockdown were placed on ice and washed to remove residual media and debris with tris-buffered saline (TBS), and then lysed using RIPA Lysis and Extraction Buffer. The protein concentration was determined using Qubit™ Protein Assay Kit reagents on a Qubit 2.0 fluorimeter device. At the next stage, electrophoretic separation of proteins from the lysates was carried out in a polyacrylamide gel using cassettes. An equal amount of protein from each sample lysate was mixed with 2× Laemmli sample buffer and boiled at 95°C for 5 min, then added to the gel pockets. The Precision Plus Protein WesternC standards molecular weight markers for chemiluminescence analysis and Precision Plus Protein All Blue standards for colorimetric analysis were added to separate pockets. Protein separation in Tris/Glycine buffer was carried out at a constant voltage of 150 V for one hour at room temperature. After electrophoresis, proteins were transferred to a nitrocellulose membrane at 20 mA overnight at a temperature of 4°C. The efficiency of transfer was assessed by staining the proteins on the membrane in a Ponceau S dye solution for 5 min. Next, the membrane was washed three times with TBST buffer, then blocked with EveryBlot Blocking Buffer for 5 min at room temperature. Next, the membrane was incubated in a solution of primary antibodies overnight at 4°C on a shaker in slow mode. The dilution of primary polyclonal antibodies to PLXNA2 was 1:1000, and primary beta-actin (ACTB) Monoclonal Antibody—1:10,000. After incubation with primary antibodies, the membrane was washed three times with TBST and then incubated for one hour at room temperature along with the secondary antibodies Goat Anti-Rabbit IgG and Goat Anti-Mouse IgG diluted 1:3000 in a solution of 5% skim milk in TBST conjugated to horseradish peroxidase HRP1. All antibody incubations were performed on an ELMI SkyLine Shaker S-4 swinging platform at low speed, approximately 40–50 revolutions per minute, in an airtight container to minimize evaporation. After incubation, the membrane was visualized using the Clarity™ Western ECL Substrate chemiluminescent kit according to the manufacturer’s instructions. The luminescence of the bands was visualized using the ChemiDoc XRS+ gel documentation system. Densitometry of the bands was carried out using the ImageLab 6.0 program (Bio-Rad Laboratories, Inc., Hercules, CA, USA).

### 2.5 Immunocytochemistry

The DTIC-treated SK-MEL-2 and A375 *PLXNA2* knockdown melanoma cells were washed, fixed with 10% formaldehyde, and permeabilized with 0.5% Triton X-100 for 10 min at room temperature. Cells were then incubated with the primary rabbit polyclonal antibody PLXNA2 at a concentration of 1:50 with 10% FBS at 4°C overnight. As secondary antibodies, the goat anti-rabbit antibodies Alexa Fluor 488 IgG (H + L) were used at a dilution of 1:200 for one hour at room temperature in the dark. Nuclei were counterstained with 1 μg/mL DAPI for 15 min. Cells were counted in at least 10 fields of view using a Floid Cell Imaging Station. Cell nuclei were stained blue, and the cytoplasm of cells expressing PLXNA2 was stained green. In each sample, the average percentage of cells stained green was calculated, which was taken as the level of expression of a given molecule.

### 2.6 Immunohistochemistry

The protocol for the examination of formalin-fixed, paraffin-embedded (FFPE) biopsies of melanocytic neoplasms collected from patients received approval from the Local Ethics Committee of Krasnoyarsk State Medical University (protocol No. 70/2016, issued on 06 June 2016).

The samples of primary melanomas (n = 35) and benign melanocytic tumors (n = 7) were kindly provided by the Krasnoyarsk Regional Pathologic Anatomy Bureau (Krasnoyarsk, Russia), where diagnosis and key histopathological features were established by a certified pathologist.

Immunohistochemistry was performed using the Mouse and Rabbit Specific HRP/AEC IHC Detection Kit—Micropolymer. For this purpose, the FFPE sections were deparaffinized in 100% xylene and dehydrated in ethanol gradients, then washed twice in PBS (10 mM/L) at pH 7.4. Unmasking was done in citrate buffer (pH 6.0) at 103°C for 30 min. Primary rabbit polyclonal antibodies to PLXNA2 were used at a dilution of 1:250. Slides were incubated for one hour at room temperature. Immunostaining was visualized using the dye 3-Amino-9-ethylcarbazole (AEC Single Solution), supplied as a ready-to-use single solution in a reagent kit. Negative controls were processed similarly but without the primary antibody. Positively stained cells were evaluated using an Olympus BX-41 microscope and an Infinity 2 Lumenera camera. Images were analyzed using Infinity Capture and Infinity Analyze software (version 6.5.2, Lumenera Corporation, Ottawa, ON, Canada). The slides were viewed at a magnification of ×400, and five fields were randomly selected for further analysis of antigen expression. Antigen expression was taken to correspond to the average value of the IHC-coefficient determined in each field of view, which was calculated as the sum of the proportions (%) of cells with intense immunovisualization, an average degree of staining, and weakly staining.

### 2.7 Real-Time Quantitative Reverse Transcription PCR Analysis (Real-Time qRT-PCR)

Total RNA isolation from SK-MEL-2 and A375 melanoma cells was carried out using the diaGene reagent kit, according to the manufacturer’s protocol. Next, a reverse transcription reaction was performed using the MMLV RT kit according to the manufacturer’s protocol. For this purpose, to each 3 μL sample of RNA solution, a random decanucleotide primer of 1.5 μL and a reaction mixture of 5.5 μL, consisting of 1 μL dNTP mixture, 1 μL DTT (1,4-dithiothreitol), 2 μL 5× first standard buffer, 0.5 μL of MMLV reverse transcriptase and 1 μL of nuclease-free water was added. The sample was incubated at 40°C for 50 min, and then the reaction was stopped by heating for 10 min at 70°C. The amplification reaction of each sample was carried out in a reaction mixture with a total volume of 20 μL for each sample, which consisted of 2 μL of cDNA, 1 μL of primers, 8 μL of a 2.5-fold reaction mixture for PCR in the presence of ROX, 1.2 μL of magnesium chloride solution and 6.8 μL of nuclease-free water. It was performed on a StepOneTM thermal cycler with a thermal cycling protocol: 1 cycle 50°C–2 min, 95°C–10 min, then 40 cycles of 95°C–15 s, 60°C–1 min. Kits used to determine mRNA expression: ITGA2, ITGA5, ITGA6, ITGB1, ITGB3, ITGB5, ITGB8, PLXNA2, FPFR2, PIK3R1, СYP1A1, CYP1A2, CYP2E1. HPRT1, ACTB and GAPDH were used as endogenous controls.

The obtained data were analyzed by calculating the expression level using the “ΔΔCT-method” [32].

### 2.8 Cell Cycle Assay

After exposure of the SK-MEL-2 and A375 melanoma cells to DTIC in concentration 1.2 mmol/L followed by transfection of siRNA to PLXNA2, the cells were trypsinized (0.25% Trypsin-EDTA solution), then fixed with 10% formaldehyde, permeabilized with 0.1% Triton X-100 and stained with a fluorescein-conjugated monoclonal antibody to Ki-67 at a concentration of 1:100 for four hours at room temperature, followed by the incubation with 100 μg/mL PI (Invitrogen; Thermo Fisher Scientific, Inc., Carlsbad, CA, USA) for an additional 20 min in a dark. The proportion of cells in each phase of the cell cycle was determined using a Cytomics FC-500 flow cytometer and CXP software (version 2.2). Gating strategies for determining the G0 cell population were as described by Kim, Sederstrom, 2015 [33]. The experiment was done in three replicates.

### 2.9 Annexin V/7-AAD Apoptosis Assay

To determine the proportion of alive, apoptotic, and necrotic cells, SK-MEL-2 and A375 melanoma cells were stained with Annexin V-FITC-conjugated and 7-AAD, according to the manufacturer’s protocol of the Annexin V-FITC/7-AAD Apoptosis Assay Kit. Briefly, cell suspension samples at a concentration of 5 × 10^5^ were incubated on ice with the indicated dyes in the binding buffer provided in the kit for 15 min in the dark. Next, the staining result was analyzed on a Cytomics FC-500 flow cytometer. Distribution of cells was as follows: viable cells (annexin-V^−^/7AAD^−^), early apoptotic cells (annexin-V^+^/7AAD^−^), late apoptotic/necrotic cells (annexin-V^+^/7AAD^+^) and necrotic cells/cellular debris (annexin-V^−^/7AAD^+^). Distribution was performed automatically on the CXP cytofluorimeter software, and the percentage of cells in each category.

### 2.10 Statistical Analysis Methods

Statistical analysis was carried out using the statistical analysis software package Statistica 7.0 (StatSoft, Tulsa, OK, USA). A Kolmogorov-Smirnov test was used to assess the normality of data distribution. Then, a nonparametric Mann-Whitney U test was used for pairwise comparisons of data between study groups. Correlation relationships were identified using Spearman’s rank correlation analysis. Differences were considered statistically significant at a *p-*value of <0.05. Data is presented as means and standard errors of the mean (mean ± SEM).

## 3 Results

### 3.1 Melanoma Cells’ Ability to Metabolize DTIC

**Figure 1:**
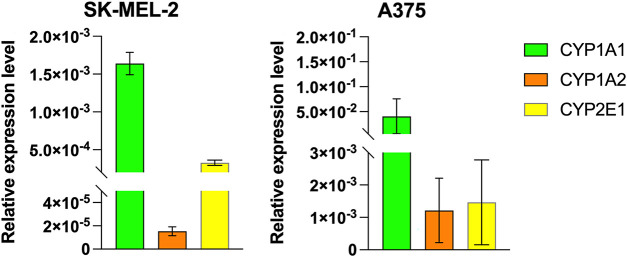
Cytochrome P1A1, P1A2, P2E1 expression in SK-MEL-2 and A375 melanoma cells.

Canonically, DTIC is metabolized in the liver by cytochromes CYP1A1, CYP1A2, CYP2E1 [34]. Besides, extrahepatic metabolism of DTIC has been proven and is mediated by cells largely through cytochrome CYP1A1 and CYP1A2, and to a limited extent through CYP2E1 [34,35]. To determine how closely our *in vitro* experiments match the pharmacokinetic effects of dacarbazine that are present *in vivo*, we analyzed the mRNA expression levels of the aforementioned proteins in SK-MEL-2 and A375 melanoma cells using real-time qRT-PCR. Our results confirmed that all types of cytochromes are expressed in SK-MEL-2 and A375 melanoma cells, demonstrating the potential of possible drug metabolism *in vitro* (Fig. 1).

### 3.2 DTIC-Treated Melanoma Cells Characterized by Enhanced Adhesive Capacities to Extracellular Matrix Proteins

**Figure 2:**
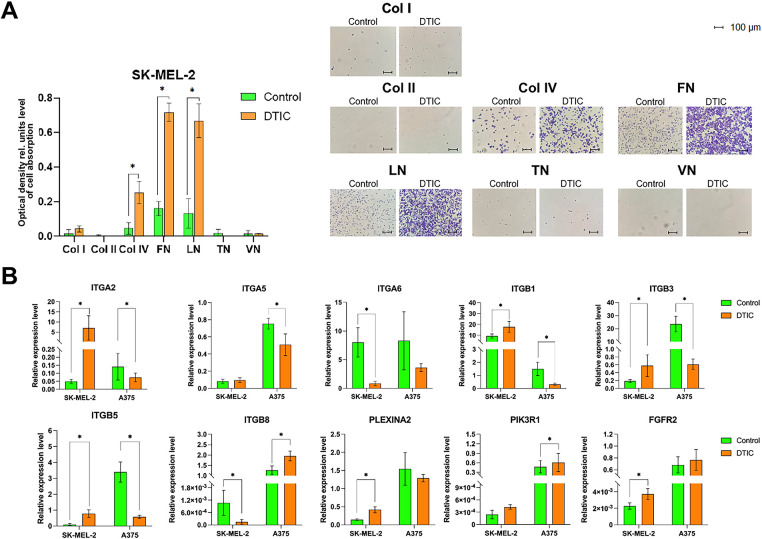
Results of a study of melanoma cell adhesion to various extracellular matrix components and the expression of adhesion-related genes after DTIC treatment. (**A**) Adhesion of DTIC-treated SK-MEL-2 melanoma cells to various extracellular matrix components. Data are presented as histograms and representative micrographs. Histograms depict the quantitative assessment of adhesive cells, reflecting the number of cells that remained attached to each ECM component following a standardized adhesion assay based on spectrophotometry. Micrographs illustrate the pattern of cellular attachment on different matrix substrates, scale bar is 100 μm. Abbreviations: Col I–Collagen type I, Col II–Collagen type II, Col IV–Collagen type IV, FN–Fibronectin, LN–Laminin, TN–Tenascin. (**B**) Results of assessing the expression level of molecules regulating adhesion processes using the real-time qRT-PCR method in melanoma cells SK-MEL-2 and A375. *Statistically significant differences for *p* < 0.05.

A colorimetric adhesion assay was applied to determine the adhesion pattern of DTIC-treated melanoma cells. Indeed, among several extracellular matrix proteins, collagen type I, collagen type II, collagen type IV, fibronectin, laminin, tenascin, vitronectin, DTIC-treated SK-MEL-2 were characterized by a 5.6-fold increase in adhesion to collagen type IV (*p* = 0.0495) and by a 4.6-fold increase to fibronectin and laminin (Fig. 2A).

### 3.3 DTIC-Treated Melanoma Cells Exert an Altered Expression Pattern of Adhesive Molecules

As we revealed that DTIC can modify the adhesive properties of cancer cells, an evaluation of the mRNA expression profile of adhesion molecules was carried out. We selected differentially expressed adhesion molecules based on our previous results on the transcriptomic profiling of DTIC-treated melanoma cells [14].

Significant alterations in gene expression (*p* = 0.0495) were observed in SK-MEL-2 melanoma cells exposed to DTIC compared to the control. Specifically, a substantial increase in the expression of the *ITGA2*, *ITGB1*, *ITGB3*, and *ITGB5* genes was noted, with fold changes of 142.0, 1.9, 3.1, and 8.3, respectively. Furthermore, the expression of the *PLXNA2* gene increased by 2.9-fold, and *FGFR2* by 1.6-fold. Conversely, the expression of the *ITGA6* and *ITGB8* genes was significantly reduced by 9.4 and 7.3-fold, respectively, compared to the control (*p* = 0.0495 for all instances).

An mRNA analysis of A375 melanoma cells revealed a decrease in the expression levels of *ITGA2, ITGB1, ITGB3*, and *ITGB5* by 1.9, 1.5, 4.7, and 5.9-fold, respectively, following DTIC treatment compared to the control (*p* = 0.0495 for all instances). Notably, *ITGB8* mRNA levels were 1.6-fold higher, and an increase in *PIK3R1* gene expression by 1.3-fold was observed in DTIC-treated A375 melanoma cells compared to the control (Fig. 2B).

### 3.4 PLXNA2 Is Overexpressed in Melanoma Versus Melanocytic Nevi Samples

As PLXNA2 was found to be dysregulated in melanoma cells under DTIC treatment, we carried out an immunohistochemical study to unveil whether PLXNA2 expression was dysregulated in melanoma in comparison to benign melanocytic neoplasms—melanocytic nevi.

A total of 35 formalin-fixed, paraffin-embedded (FFPE) tissue samples from malignant melanoma and seven benign melanocytic neoplasms (nevi) from Caucasian patients were subjected to immunocytochemical assay. Patient’s demographic data is presented in Table 1.

**Table 1:** Clinical and histopathological characteristics of melanoma samples used for the study of PLXNA2 expression by immunohistochemistry.

No	Sex	Age	Pathological Variant	Tumor Thickness According to Breslow, mm	Growth Type	Type of Lymphocytic Infiltrate	AJCC	PLXNA2 IHC-Coefficient
1	Female	86	SSM	5.7	Vertical	Brisk	IIB	1.19
2	Female	38	SSM	2.0	Vertical	Nonbrisk	IIA	2.35
3	Male	80	SSM	6.2	Vertical	Brisk	IIC	2.96
4	Female	73	SSM	0.7	Radial	Аbsent	IB	2.10
5	Male	73	NM	8.0	Vertical	Nonbrisk	IIC	0.61
6	Female	59	NM	1.9	Vertical	Brisk	IIA	0.95
7	Female	61	SSM	1.3	Vertical	Аbsent	IB	0.79
8	Male	56	SSM	0.9	Radial	Brisk	IA	0.98
9	Female	66	SSM	4.4	Vertical	Brisk	IIB	1.02
10	Male	50	LM	5.8	Vertical	Brisk	IIC	2.71
11	Female	54	SSM	3.6	Vertical	Nonbrisk	IIB	1.35
12	Male	57	LM	4.9	Vertical	Brisk	IIC	0.88
13	Male	66	SSM	3.4	Vertical	Brisk	IIA	1.39
14	Male	67	SSM	4.2	Radial	Nonbrisk	IV	2.12
15	Female	46	SSM	1.7	Radial	Nonbrisk	IB	1.93
16	Female	58	SSM	3.2	Vertical	Brisk	IIA	0.77
17	Female	59	SSM	3.3	Vertical	Brisk	IIA	1.01
18	Female	90	NM	5.4	Vertical	Nonbrisk	IIIC	0.89
19	Male	53	NM	3.5	Vertical	Brisk	IIIC	0.95
20	Female	36	LM	3.6	Vertical	Brisk	IV	2.08
21	Female	34	SSM	4.6	Vertical	Аbsent	IIIC	1.35
22	Female	51	SSM	4.3	Vertical	Brisk	IV	0.92
23	Male	50	–	–	–	–	–	1.49
24	Male	51	–	–	–	–	–	0.99
25	Unknown	77	–	–	–	–	–	1.06
26	Female	53	–	–	–	–	–	1.97
27	Male	80	–	–	–	–	–	2.12
28	Unknown	–	–	–	–	–	–	1.47
29	Unknown	–	–	–	–	–	–	1.00
30	Unknown	–	–	–	–	–	–	1.42
31	Unknown	–	–	–	–	–	–	2.36
32	Unknown	–	–	–	–	–	–	2.93
33	Unknown	–	–	–	–	–	–	1.81
34	Unknown	–	–	–	–	–	–	1.39
35	Unknown	–	–	–	–	–	–	1.28

Note: Unknown or unexplored data were reported as “-”; SSM, Superficial spreading melanoma; NM, Nodular melanoma; LM, Lentigo melanoma; AJCC, American joint committee on cancer; FFPE, Formalin-fixed paraffin-embedded; PLXNA2, Plexin A2; IHC, Immunohistochemistry.

Lentigo melanoma clinical type was presented in 13.6% of cases, superficial spreading melanoma and NM 68.2% and 18.2% of cases, respectively.

Nevus samples were obtained from patients aged 20 years to 65 years old, of whom 83.3% were women (the average age was 40.0 years ± 9.15 years) and 16.7% were men (the only patient aged 23 years).

PLXNA2 immunovisualization demonstrated cytoplasmic staining of tumor and nevus cells with various degrees of intensity both in melanomas and melanocytic nevi. Additionally, epidermal keratinocytes showed high to moderate levels of cytoplasmic staining. Moreover, PLXNA2 was also expressed in endothelial cells.

We have not established any correlation between the expression of PLXNA2 and patients’ age or Breslow tumor thickness. Furthermore, no differences in PLXNA2 expression were observed depending on the sex of melanoma patients, tumor growth pattern, type of lymphocytic infiltration, or American Joint Committee on Cancer (AJCC) stage.

**Figure 3:**
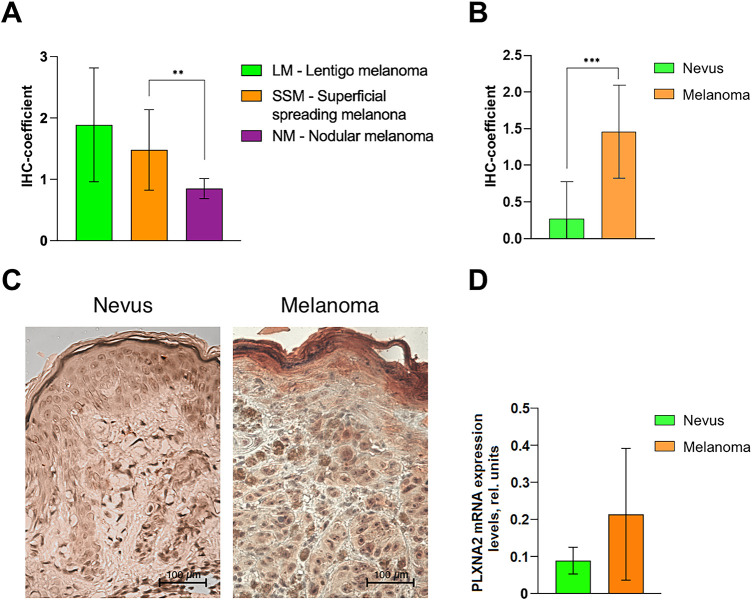
PLXNA2 expression in cutaneous melanoma and benign melanocytic nevi. (**A**) PLXNA2 expression in melanoma patients’ samples due its clinical type. (**B**) PLXNA2 expression in melanocytic nevi and melanoma according to the results of immunohistochemistry. (**C**) Melanocytic nevus and melanoma tissue stained with antibodies to PLXNA2, ×200 magnification. Low-intensity weak reddish staining localized in the cytoplasm of nevus cells is observed in melanocytic nevi. Intense cytoplasmic staining of cell cytoplasm in melanoma. (**D**) PLXNA2 expression in melanocytic nevi and melanoma based on real-time qRT-PCR results. **Statistically significant differences for *p* < 0.01 and ***for *p* < 0.001.

However, we found that the expression of PLXNA2 was 56% lower (*p* = 0.0028) in patients with nodular melanoma (NM) as compared to patients with superficial spreading melanoma (SSM), but did not differ from those with lentigo melanoma (LM). PLXNA2 expression also did not differ between patients with LM and SSM (Fig. 3A).

The IHC-coefficient of PLXNA2 expression was 0.27 ± 0.19 in melanocytic nevi, whereas the PLXNA2 IHC-coefficient was 1.47 ± 0.1 (*p* = 0.0003) in melanoma tissue, a 5.44-fold difference (Fig. 3B,C).

To further analyze whether alterations in *PLXNA2* expression are associated with changes in mRNA expression levels, we studied lesioned tissue—melanoma and melanocytic nevi samples kept in an RNA-stabilized solution. Seven samples were examined: four nevi and three melanomas. The mean age of patients with nevi was 36.0 years ± 2.74 years, and there were two men and two women. The clinical data of the melanoma patients are presented in Table 2.

**Table 2:** Clinical and histopathological characteristics of melanoma samples used for PLXNA2 expression study by real-time qRT-PCR.

No	Sex	Age	Pathological Variant	Tumor Thickness According to Breslow, mm	Localization
1	Male	73	NM	2.0	Shoulder
2	Female	75	SSM	1.1	Abdomen
3	Male	62	NM	3.0	Back

Note: SSM, Superficial spreading melanoma; NM, Nodular melanoma.

A real-time qRT-PCR expression analysis did not reveal differences between mRNA levels of *PLXNA2* in the tissues of melanomas and benign nevi (which may be associated with both the small sample set and the predominance of the nodular form of melanoma, characterized by the lowest values of *PLXNA2* expression among other clinical and morphological forms). However, there is still an evident tendency towards an increase in the average value of PLXNA2 expression in melanomas compared to melanocytic nevi, which is consistent with our results obtained by the IHC method (Fig. 3D).

### 3.5 PLXNA2 Knockdown in DTIC-Treated Melanoma Cells

**Figure 4:**
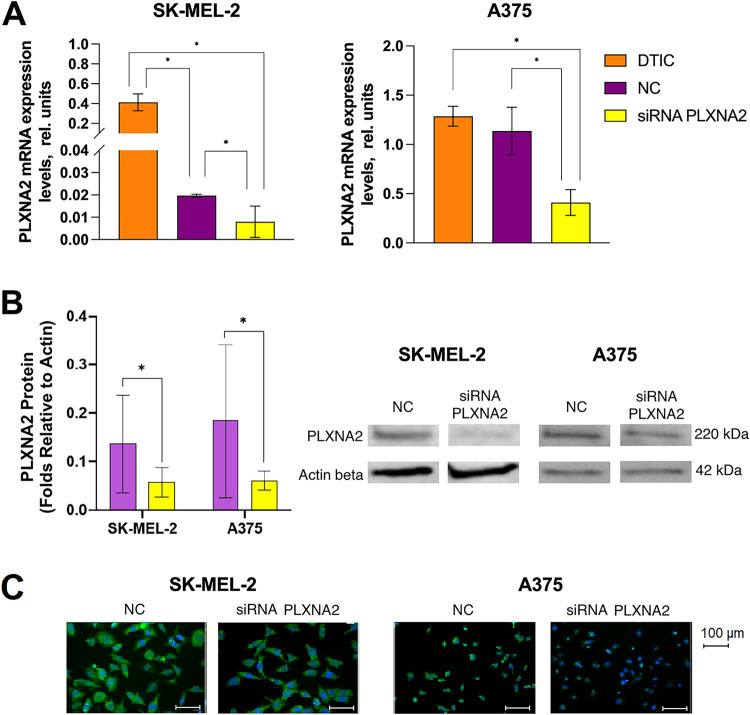
siRNA-mediated *PLXNA2* knockdown in SK-MEL-2 and A375 melanoma cells. (**A**) PLXNA2 mRNA expression. (**B**) PLXNA2 protein expression. Photographs of the membrane stained with antibodies to PLXNA2 and ACTB. (**C**) Immunocytochemical imaging of antisense oligonucleotide transfection efficiency in melanoma cells. Cell nuclei are stained blue, PLXNA2 expression is visualized as cytoplasmic green staining, scale bar is 100 μm. *Statistically significant differences for *p* < 0.05.

PLXNA2 is implicated in cancer cell proliferation and angiogenesis [36] and was found to be overexpressed in melanoma cells. Next, we performed *PLXNA2* knockdown in DTIC-treated melanoma cells to investigate its impact on cell survival and cell cycle progression. Transfection of PLXNA2 specific siRNA in DTIC-treated melanoma cells resulted in a 2.2-fold decrease (*p* = 0.0495) in its mRNA expression in SK-MEL-2 cells as compared to the negative control, which is 46.3-fold decreased compared to DTIC-treated melanoma cells without siRNA transfection. Transfection of PLXNA2 siRNA into DTIC-treated A375 melanoma cells resulted in a 2.8-fold decrease (*p* = 0.0495) in *PLXNA2* expression compared to the negative control, and a 3.1-fold decrease compared to DTIC-treated cells (Fig. 4A).

Immunoblotting showed that PLXNA2 protein levels decrease in DTIC-treated PLXNA2-melanoma cells, showing a 3.1-fold reduction in SK-MEL-2 cells and a 3.2-fold reduction in A375 cells compared to the negative control (*p* = 0.0495) (Fig. 4B). Similar data were obtained via the immunocytochemical staining of the melanoma cells (Fig. 4C).

### 3.6 Evaluation of Cell Cycle Alterations in Melanoma Cells Exposed to DTIC Followed by PLXNA2 Knockdown

**Figure 5:**
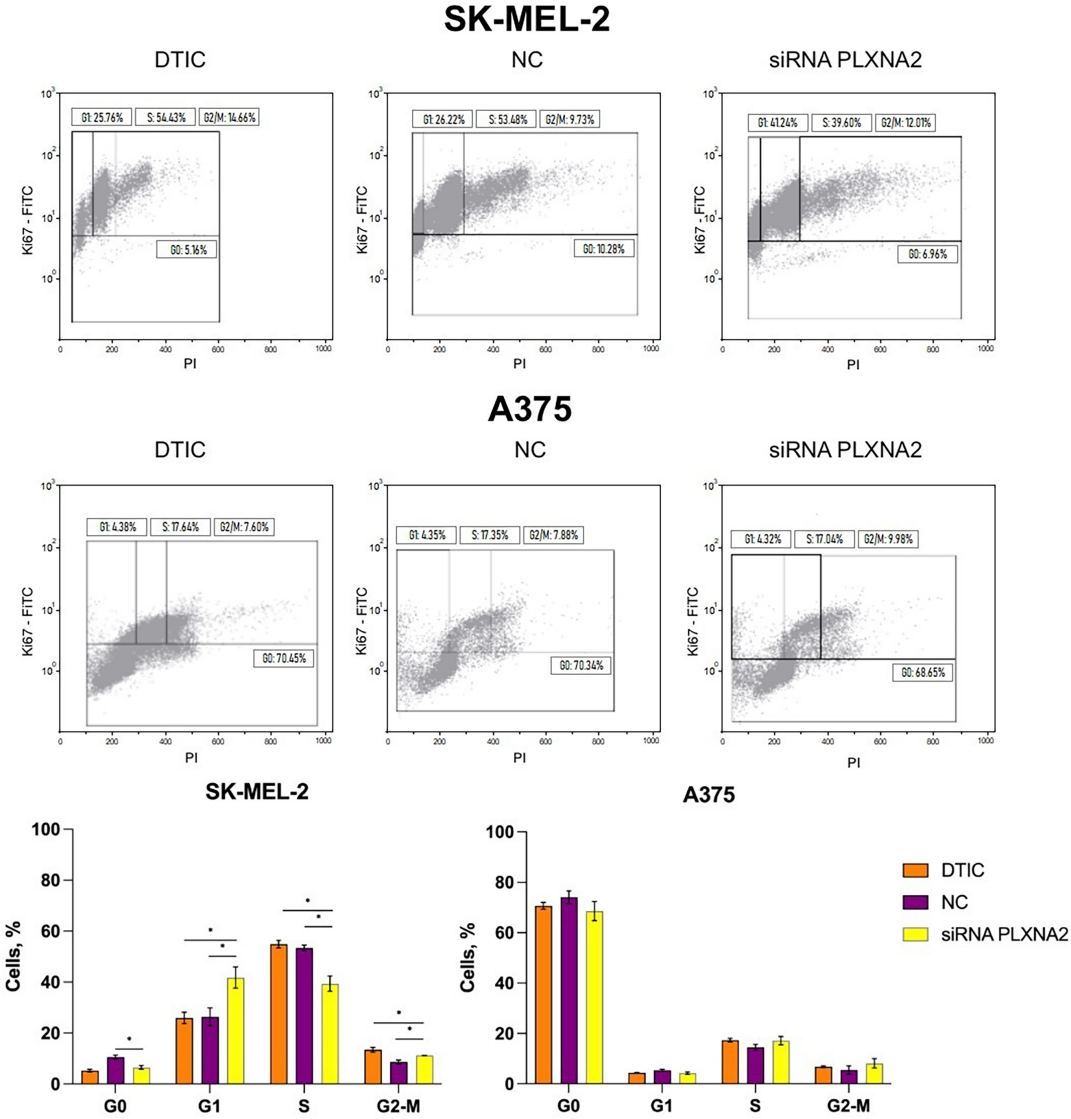
Cell cycle phase analysis distribution of melanoma cells. *Statistically significant differences for *p* < 0.05.

Based on flow cytometry analysis, the combination of *PLXNA2* knockdown with DTIC therapy in SK-MEL-2 melanoma cells resulted in 1.6-fold decrease (*p* = 0.0495) in the proportion of G0 cells compared to the negative control (from 10.5% to 6.5%), a 1.6-fold increase (*p* = 0.0495) in the proportion of cells in the G1 phase compared to the negative control (from 26.4% to 41.8%), a 1.6-fold increase compared to DTIC-treated cells (from 25.9% to 41.8%); a 1.4-fold decrease (*p* = 0.0495) in the percentage of cells in the S phase compared to the negative control (from 53.4% to 39.4%) and a 1.4-fold decrease compared to cells with DTIC monotherapy (from 54.9% to 39.4%). The proportion of melanoma cells residing in the G2-M phase was increased 1.3-fold (*p* = 0.0495) compared to the negative control (from 8.7% to 11.2%). Notably, in A375 melanoma cells, combined treatment using DTIC along with *PLXNA2* knockdown did not lead to statistically significant changes in the cell cycle (Fig. 5).

### 3.7 PLXNA2 Knockdown Induces Apoptosis in DTIC-Treated Melanoma Cells

Based on the flow cytometry analysis, the proportion of viable SK-MEL-2 melanoma cells remained unchanged following DTIC treatment combined with *PLXNA2* knockdown, as compared to both the negative control and DTIC monotherapy. However, in A375 melanoma cells, DTIC treatment followed by PLXNA2 siRNA transfection showed no difference in the percentage of live cells compared to the negative control, but resulted in a 4.3% reduction (from 95.3% to 91.0%) in the percentage of alive cells compared to cells treated only with DTIC (*p* = 0.0495).

*PLXNA2* knockdown did not alter apoptosis levels in SK-MEL-2 melanoma cells compared to the negative control, but resulted in a 1.7-fold increase (*p* = 0.0495, from 2.8% to 4.7%) compared to DTIC-treated cells.

The proportion of cells in late apoptosis/necrosis decreased 1.5-fold (*p* = 0.0495, from 3.5% to 2.3%) compared to the negative control, but remained unchanged in comparison to DTIC-treated cells.

**Figure 6:**
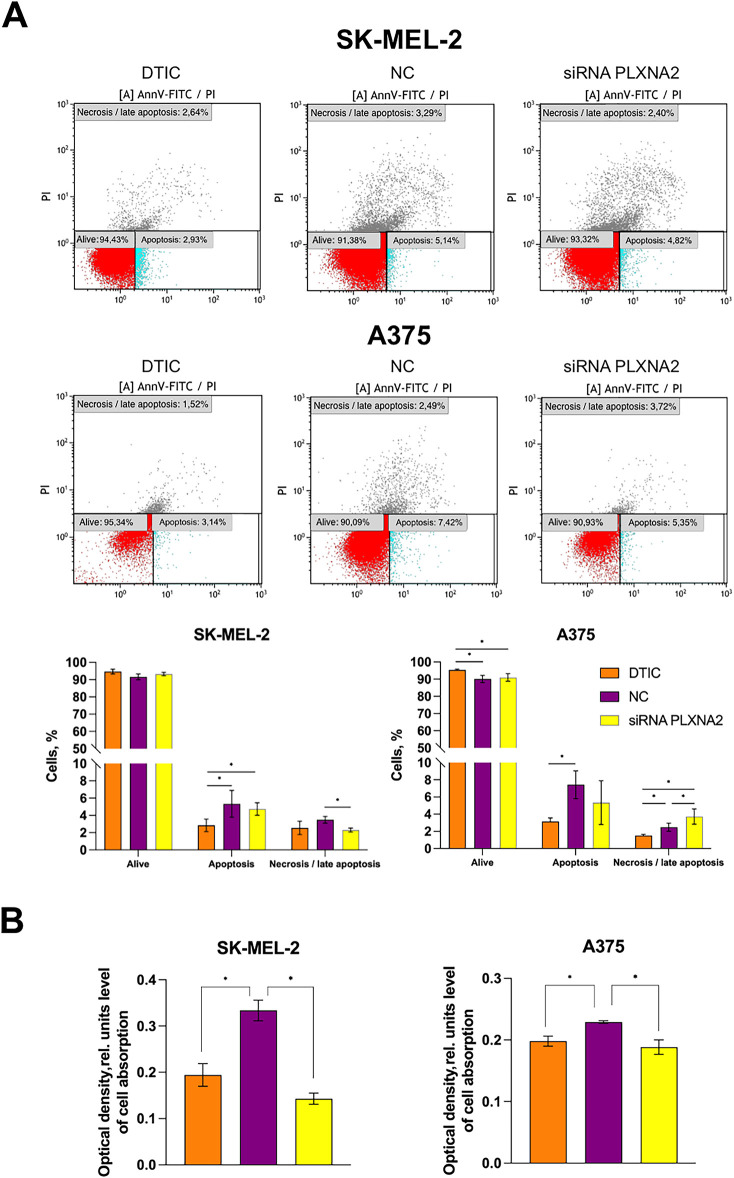
Results of a study of apoptosis and adhesion to fibronectin for melanoma cells after DTIC treatment and PLXNA2 knockdown. (**A**) Melanoma cell apoptosis under DTIC treatment and PLXNA2 knockdown. (**B**) The Study of melanoma cell adhesion to fibronectin by spectrophotometry. *Statistically significant differences for *p* < 0.05.

In A375 cells, the percentage of apoptotic cells was not altered in DTIC + *PLXNA2* knockdown treatment group compared to either the negative control and to cells treated with DTIC only. However, the percentage of late apoptotic/necrotic A375 cells increased (*p* = 0.0495) in 1.5-fold compared to the negative control (from 2.5% to 3.7%) and 2.5-fold as compared to cells treated with DTIC only (from 1.5% to 3.7%) (Fig. 6A).

### 3.8 PLXNA2 Knockdown Induces Adhesion to Fibronectin of DTIC-Treated Melanoma Cells

The combination of DTIC treatment and *PLXNA2* knockdown in SK-MEL-2 and A375 melanoma cells resulted in a 2.3-fold decrease (*p* = 0.0495) in adhesion to fibronectin compared to the negative control for SK-MEL-2 cells and in a 1.2-fold decrease in A375 cells. However, no significant difference was observed in adhesion levels of SK-MEL-2 and A375 cells as compared cells treated with DTIC only (Fig. 6B).

## 4 Discussion

DTIC is monofunctional alkylating agent with cytotoxic activity that is applied for treatment of malignant tumors [26]. However, besides weak proapoptotic activity through DNA damage induction, DTIC is characterized by evident resistance to its action by cancer cells. Previously, we determined that DTIC induces the transfer of melanoma cells to the G0 phase of the cell cycle [12]. Therefore, in the present study DTIC was applied to increase the number of G0-positive cell population in melanoma cells to study cell cycle-related cancer drug resistance mechanisms.

Melanoma cells were treated with DTIC for 72 h followed by a 48-h culturing to eliminate apoptotic cells. As mentioned above, DTIC induces G0/G1 cell cycle arrest [12] that corresponds to cells acquiring a quiescent/senescent state. Cancer cell quiescence is regulated by interactions between these cells residing in G0 and the extracellular matrix [37]. Indeed, the present study demonstrated an increased interaction between DTIC-treated melanoma cells and collagen type IV, fibronectin and laminin. Recent studies have shown that metastasis initiating colorectal cancer cells interact actively via integrin β6 with fibronectin to reprogram their phenotype [38]. Moreover, breast cancer cells cultured on collagen and fibrinogen gels exhibited G0/G1 arrest which was interpreted as obtaining quiescent state [39]. The binding of breast cancer cells to collagen type I and fibronectin decreased their sensitivity to cisplatin, doxorubicin and mitoxantrone [40]. These results, when taken together, suggest that the sensing of the extracellular matrix by cancer cells is crucial for the cell-cycle state and for acquiring cell-cycle-dependent drug resistance. Moreover, we propose that the primary cause of elevated adhesion strength can be canonical increase in highly specific complementary binding sites (adhesion molecules) to precisely defined amino acid sequences of ECM components (like the RGD sequence in fibronectin).

These compounds’ strength and stability are achieved by a variety of molecular forces, including hydrogen bonds between polar groups at the receptor and ligand binding sites, van der Waals forces (which are non-specific but quite numerous and contribute to the overall stability of binding), and electrostatic interactions between charged amino acid residues at the receptor and ligand binding sites, if the latter have a complementary charge [41,42]. Cancer cells by themselves can produce and release extracellular matrix proteins to regulate microenvironment stiffness that, in turn, coordinates their mechanobiology including cytoskeleton tension modification and the activation of mechanotransduction signaling pathways. Aside from this, cancer cells may accommodate collagen cross-links by lysyl hydroxylases [43]. These changes maintain a tumor’s growth and progression by establishing dynamic feedback between cancer cells and their microenvironment. However, integrins are considered as key receptors for interactions with extracellular matrix providing intracellular downstream mechanotransductive signaling.

Subsequently, mRNA expression of adhesion proteins in cells surviving under cytotoxic treatment was analyzed to elucidate the molecular mechanisms underlying the communication between DTIC-treated melanoma cells and extracellular matrix proteins. Surprisingly, most of the integrins’ expression we studied was down-regulated in DTIC-treated melanoma cells: *ITGA2, ITGA5, ITGA6, ITGB1, ITGB3, ITGB5* and *ITGB8* whereas signaling molecules mediating focal adhesion were up-regulated: *PLXNA2, PIK3R1, FGFR2*. Interestingly, another injurious agent in the form of ultraviolet irradiation has been shown to induce *ITGA6* down-regulation in melanocytes that corresponded to the increase in their adhesion to laminins. This alteration supported the resistance of melanocytes to apoptosis induced by ultraviolet irradiation [44]. Another study determined that enhanced adhesion of both normal and malignant cells to laminin made them resistant to various apoptotic stimuli [45]. Thus, cytotoxic therapeutic interventions may result in a melanoma cell’s autonomous behavior, phenotypic reprogramming, and survival.

Together with that, we identified a decrease in adhesion to fibronectin by DTIC+ *PLXNA2* knockdown melanoma cells. Previous studies have shown that quiescent cancer cells secrete extracellular matrix proteins to support adhesion which is necessary for their survival. Conversely, fibronectin depletion suppressed cancer growth [46,47]. These data are consistent with our results. Indeed, the percentage of apoptotic cells was lower in DTIC+ *PLXNA2* knockdown melanoma cells that were characterized by diminished adhesion to fibronectin.

Furthermore, we observed the dysregulation of focal adhesion signaling molecules. Among them, PLXNA2 showed increased expression in SK-MEL-2 melanoma cells, but decreased in A375 cells. This contradictory response to cytotoxic stress is most likely due to the specific genomic and epigenomic features of each cell line. Therefore, DTIC treatment may induce diverse signal pathways in these cells, leading to multidirectional *PLXNA2* expression [46]. At the same time, immunohistochemistry analysis of PLXNA2 expression in melanoma cells compared to benign melanocytic nevi showed that the gene’s increased expression in melanomas may correspond to its role in melanoma progression.

Therefore, an *in vitro* assay was conducted to evaluate key cellular characteristics following *PLXNA2* downregulation in DTIC-induced quiescent melanoma cells, which exhibit distinct constitutive profiles, particularly with respect to *PLXNA2* expression levels.

However, the reduction of *PLXNA2* levels in DTIC-treated melanoma cells induced apoptosis in both surviving SK-MEL-2 and A375 melanoma cells. This suggests that altered plexin signaling may modulate apoptotic pathways, potentially involving changes in the balance of pro- and anti-apoptotic proteins or alterations in receptor-mediated cell death signaling. Neufeld G. et al. reported that *PLXNA2* suppression resulted in cell cycle arrest in the G2/M phase, accompanied by increased expression of senescence-associated β-galactosidase, as well as decreased AKT phosphorylation and increased p38MAPK phosphorylation. Furthermore, they found that the pro-proliferative effect of PLXNA2 is mediated by apoptosis-related FARP2 and FYN, as well as a GTPase-activating protein (GAP) domain located within the intracellular domain of PLXNA2 [36].

Moreover, in a study on immortalized transformed keratinocytes, PLXNA2 interacted with KIAA1199, also known as CEMIP (cell migration-inducing protein), and protected cells from Semaphorin 3A (Sema3A)-induced apoptosis. This occurred, at least in part, due to stabilization of the epidermal growth factor receptor (EGFR) and the enhancement of its signaling activity [48].

*PLXNA2* knockdown in cells treated with the alkylating cytotoxic agent DTIC did not affect the percentage of G0-positive cells. However, in A375 melanoma cells, treatment with the cytotoxic agent followed by *PLXNA2* knockdown increased the percentage of cells residing in the G1 phase of the cell cycle and decreased the percentage of cells in the S phase, indicating cell cycle arrest in G1. The mechanism underlying PLXNA2’s effect on cell cycle progression in A375 cells might involve alterations in the expression or activity of cell cycle regulators, such as cyclins or cyclin-dependent kinases. Previous data demonstrated that PLXNA2 can stimulate cell cycle progression via activation of oncogene Fyn and Cdk5: stimulation by semaphorins led to the activation of Fyn, which phosphorylates Cdk5, and Cdk5 in turn phosphorylates PIKE-A, a factor promoting growth and invasion of glioma cells [49].

Atomic force microscopy combined with single-cell force spectroscopy was used to measure the adhesion force of HeLa cells to surfaces with microcontact-printed regions in the extracellular matrix proteins collagen I and fibronectin. It was found that, with a decrease in the area of the ECM region, HeLa cells enter a special state of spatially enhanced adhesion, which is characterized by a higher adhesion force per unit area and an increased density of ligand-bound integrins [50]. Another study using atomic force microscopy found that the strength of intercellular adhesion in fibroblasts with Rac1 knockdown was higher than in fibroblasts expressing this gene [51].

**Figure 7:**
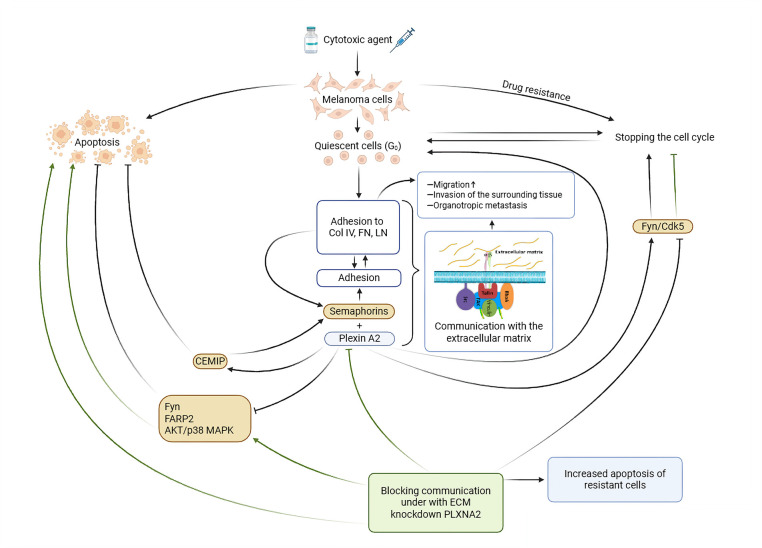
Molecular mechanisms of PLXNA2-mediated melanoma cell chemoresistance.

All potential molecular mechanisms discussed in this section, reflecting the possible mechanistic picture of the action of the PLXNA2 molecule in the implementation of chemoresistance of melanoma cells, are presented in Fig. 7. However, the study has several limitations—lack of time-course experiments, lack of *in vivo* studies, and the absence of immune microenvironment effects studies. However, the study was performed on minimal sample sizes (n = 3), resulting in a discrete distribution of *p*-values. The repeated significance level of *p* = 0.0495 in our study is due to the specificity of the Mann-Whitney rank test, which, when comparing two groups of three objects, represents the minimum threshold of statistical significance mathematically achievable under these conditions. Therefore, although our findings indicate the presence of a biological effect, they should be considered preliminary and require further validation on larger sample sizes. Future studies can focus on confirming the links between these mechanistic events and evaluating their reproducibility *in vivo*.

## 5 Conclusion

*PLXNA2* knockdown enhances apoptosis in G0-positive melanoma cells where G0 is induced by alkylating cytotoxic agents. *PLXNA2* down-regulation did not affect cell cycle progression and the percentage of cells residing in G0.

However, further investigations are needed to elucidate the extent to which modulation of focal adhesion signaling by PLXNA2 suppression can intensify cancer cell apoptosis, and to consider focal adhesion molecules targeting as additional approach to modulating cancer resistance. The obtained results suggest that *PLXNA2* can play a role in the survival of drug-induced dormant melanoma cells. *PLXNA2* down-regulation did not significantly affect the proportion of cells in G0, suggesting that it affects survival independently of the initial induction of quiescence. Taken together, these findings contribute to our understanding of the mechanisms governing drug resistance in melanoma cell quiescence. Future studies should be performed using *in vivo* models to confirm the effects obtained *in vitro*, followed by thorough analysis to identify the exact molecular mechanisms of PLXNA2-mediated effects on apoptosis and the cell cycle, and their contribution to the eradication of quiescent, drug-resistant melanoma cells. In addition, the potential of focal adhesion inhibitors as a novel therapeutic approach for non-cycling melanoma cells also needs to be explored.

## Data Availability

The data that support the findings of this study are available from the Corresponding Author, Tatiana Ruksha, upon reasonable request.

## Abbreviations

ACTB: Actin beta
CYP1A1: Cytochrome P450 family 1 subfamily A member 1
CYP1A2: Cytochrome P450 family 1 subfamily A member 2
CYP2E1: Cytochrome P450 family 2 subfamily E member 1
DMSO: Dimethyl sulfoxide
DTIC: Dacarbazine
DTT: 1,4-dithiothreitol
FBS: Fetal bovine serum
FFPE: Formalin fixed paraffin embedded
FGFR2: Fibroblast growth factor receptor 2
GAPDH: Glyceraldehyde-3-phosphate dehydrogenase
HPRT1: Hypoxanthine phosphoribosyltransferase 1
ITGA2: Integrin subunit alpha 2
ITGA5: Integrin subunit alpha 5
ITGA6: Integrin subunit alpha 6
ITGB1: Integrin subunit beta 1
ITGB3: Integrin subunit beta 3
ITGB5: Integrin subunit beta 5
ITGB8: Integrin subunit beta 8
LM: Lentigo melanoma
MMLV RT: Moloney murine leukemia virus reverse transcriptase
NM: Nodular melanoma
PIK3R1: Phosphoinositide-3-kinase regulatory subunit 1
PLXNA2: Plexin A2
qRT-PCR: Quantitative reverse transcription-PCR
RISC: RNA-induced silencing complex
SSM: Superficial spreading melanoma
TBS: Tris-buffered saline
TBST: TBS solution with the detergent Tween® 20

## Appendix A

**Equipment**

ChemiDoc XRS+ gel documentation system, Bio-Rad Laboratories, Hercules, CA, USA

CO_2_ incubator, Sanyo ElectricCo., Ltd., Kobe, Japan; MSO-5ACCO2

CXP cytofluorimeter software, Beckman Coulter, Inc., Fullerton, CA, USA

Cytomics FC-500 flow cytometer, Beckman Coulter, Inc., Brea, CA, USA

ELMI SkyLine, Elmi Ltd., Riga, Latvia, Model Shaker S-4

Floid cell imaging station, Thermo Fisher Scientific, Carlsbad, CA, USA

Infinity 2 Lumenera camera, Lumenera Corporation, Ottawa, Canada

Olympus BX-41 microscope, Olympus Corporation, Tokyo, Japan

Qubit 2.0 fluorimeter, Invitrogen by Life Technologies, Thermo Fisher Scientific Inc., Singapore

Spectrophotometer, Shvabe Photosystems, Moscow, Russia; Model EFOS-9305

StepOne™ thermal cycler, Applied Biosystems, Singapore City, Singapore

**Cell Lines**

A375, cat. no. ATCC® CRL-1619™, acquired from the Research Institute of Experimental Diagnostics and Therapy of Tumors (Moscow, Russia)

SK-MEL-2, cat. no. ATCC® HTB-68™, purchased from the BioLot (Moscow, Russia)

**Reagents**

2.5-fold reaction mixture for PCR in the presence of ROX, Syntol, Moscow, Russia; cat. no. M-431

2× Laemmli sample buffer, Bio-Rad Laboratories, USA; cat. no. 1610737

4%–20% Mini-PROTEAN^®^ TGX Stain-Free™ Protein Gels, 10 wells, 50 μL, Pkg of 10, 4%–20% precast polyacrylamide gel, 8.6 cm × 6.7 cm (W × L), Bio-Rad Laboratories, USA; cat. no. 4568094

Annexin V-FITC/7-AAD Apoptosis Assay Kit, Beckman Coulter Company, Inc., Marseille, France; cat. no. IM3614

Antibiotic-antimycotic, PanEco, Moscow, Russia

BioMaster Myco-visor kit, Biolabmix, Novosibirsk, Russia, cat. no. Myc-16S-400

Clarity™ western ECL substrate chemiluminescent kit, Bio-Rad Laboratories, USA; cat. no. 1705060

DAPI, AppliChem GmbH, Darmstadt, Germany

DiaGene reagent kit, Dia-m, Moscow, Russia; cat. no. 3317

DMEM with glutamine and glucose 4.5 g/L, PanEco, Moscow, Russia

DMSO (Dimethyl Sulfoxide), PanEco, Moscow, Russia

DTIC (Dacarbazine), Sigma-Aldrich, St. Louis, MO, USA

ECM cell adhesion array kit, colorimetric, Sigma-Aldrich, Darmstadt, Germany; cat. no. ECM540

EveryBlot blocking buffer, Bio-Rad Laboratories, Tokyo, Japan; cat. no. 1201002

FBS (Fetal Bovine Serum), Cytiva, Marlborough, MA, USA

Fibronectin, Sigma-Aldrich, St. Louis, MO, USA; cat. no. F1056-1MG

Goat Anti-Mouse IgG (H + L)-HRP Conjugate, Bio-Rad Laboratories, USA; cat. no. 1706516

Goat Anti-Rabbit IgG (H + L)-HRP Conjugate, Bio-Rad Laboratories, USA; cat. no. 1706515

Molecular weight markers Precision Plus Protein All Blue Standards, Bio-Rad Laboratories, USA; cat. no. 161-0373

Molecular weight markers Precision Plus Protein WesternC Standards, Bio-Rad Laboratories, USA; cat. no. 161-0376

MMLV RT kit reagent kit, Evrogen, Moscow, Russia; cat. no. SK021

Mouse and rabbit specific HRP/AEC IHC detection kit—Micropolymer, Abcam, Braintree, MA, USA; cat. no. ab236467

Nitrocellulose membranes, 0.45 μm, 7cm × 8.5 cm, Bio-Rad Laboratories Inc., USA; cat. no. 1620145

Ponceau S dye solution for electrophoresis (0.2%), SERVA Electrophoresis GmbH, Heidelberg, Germany; cat. no. 33427.01

Propidium iodide (PI), Invitrogen; Thermo Fisher Scientific, Inc., Carlsbad, USA

Qubit™ Protein Assay Kit, Invitrogen; Thermo Fisher Scientific Inc., Eugene, OR, USA

RIPA lysis and extraction buffer Thermo Scientific™, Thermo Scientific, Loves Park, IL, USA; cat. no. 89900

Tris-buffered saline (TBS), Bio-Rad Laboratories, Hercules, CA, USA; cat. no. 1706435

Tris/Glycine buffer for western blots and native gels, Bio-Rad Laboratories, USA; cat. no. 1610732

Triton X-100, GERBU Biotechnik GmbH, Gaiberg, Germany

Trypsin-EDTA solution (0.25%), Gibco Life Technologies, Grand Island, NY, USA

TWEEN 20, GERBU Biotechnik GmbH, Gailberg, Austria; cat. no. 2001

**Antibodies**

Fluorescein-conjugated monoclonal antibody to Ki-67 (FITC), eBioscience, Thermo Fisher Scientific, Inc., Carlsbad, USA; cat. no. SolA15

Goat anti-rabbit antibodies Alexa Fluor 488 IgG (H + L), cat. no., 1706515; Invitrogen, Thermo Fisher Scientific, Eugene, OR, USA

Primary ACTB monoclonal antibody, Invitrogen, Thermo Fisher Scientific, Loves Park, IL, USA; cat. no. 15G5A11/E2

Primary polyclonal antibodies to PLXNA2 Ab, Affinity Biosciences, Changzhou, China; cat. no. DF9760

**Kits used to determine mRNA expression**

ACTB (Hs99999903_m1, Applied Biosystems, Thermo Fisher Scientific, Carlsbad, CA, USA)

CYP1A1 (Hs01054796_g1, Applied Biosystems, Thermo Fisher Scientific, Carlsbad, CA, USA)

CYP1A2 (Hs00167927_ml, Applied Biosystems, Thermo Fisher Scientific, Carlsbad, CA, USA)

CYP2E1 (Hs00559367_ml, Applied Biosystems, Thermo Fisher Scientific, Carlsbad, CA, USA)

GAPDH (Hs02786624_g1, Applied Biosystems, Thermo Fisher Scientific, Carlsbad, CA, USA)

HPRT1 (Hs02800695_m1, Applied Biosystems, Thermo Fisher Scientific, Carlsbad, CA, USA)

PIK3R1 (Hs00933163_m1, Applied Biosystems, Thermo Fisher Scientific, Carlsbad, CA, USA)

ITGA2 (Gene ID, 3673, Ensembl, ENSG00000164171, DNA-Sintez, Moscow, Russia)

ITGA5 (Gene ID, 3678, Ensembl, ENSG00000161638, DNA-Sintez, Moscow, Russia)

ITGA6 (Gene ID, 3655, Ensembl, ENSG00000091409, DNA-Sintez, Moscow, Russia)

ITGB1 (Gene ID, 3688, Ensembl, ENSG00000150093, DNA-Sintez, Moscow, Russia)

ITGB3 (Gene ID, 3690, Ensembl, ENSG00000259207, DNA-Sintez, Moscow, Russia)

ITGB5 (Gene ID, 3693, Ensembl, ENSG00000082781, DNA-Sintez, Moscow, Russia)

ITGB8 (Gene ID, 3696, Ensembl, ENSG00000105855, DNA-Sintez, Moscow, Russia)

PLXNA2 (Gene ID, 5362, Ensembl, ENSG00000076356, DNA-Sintez, Moscow, Russia)

FPFR2 (Gene ID, 2263, Ensembl, ENSG00000066468, DNA-Sintez, Moscow, Russia)

**Reagents for transfection**

GenJect transfection reagent, cat. no. Gen39-1000p, Molekta, Moscow, Russia

Scrambled siRNA, DNA-Sintez company, Moscow, Russia, sequence of scrambled siRNA, which is a set of nucleotides in a rasssndom sequence CGA AGU UGG AGU CUG CUG UUA GUC A-3^′^ 3^′^-GCU UCA ACC UCA GAC GAC AAUCAG U

SiRNA to PLXNA2, DNA-Sintez company, Moscow, Russia, sequence of siRNA to PLXNA2 as GGU AGU GAG UGA UGC UCC UGA UCU A-3^′^ 3^′^-CCA UCA CUC ACU ACG AGG ACU AGA U
